# Simulation-guided design of serological surveys of the cumulative incidence of influenza infection

**DOI:** 10.1186/1471-2334-14-505

**Published:** 2014-09-17

**Authors:** Kendra M Wu, Steven Riley

**Affiliations:** School of Public Health, LKS Faculty of Medicine, The University of Hong Kong, Hong Kong, SAR China; Medical Research Council Centre for Outbreak Analysis and Modelling, Department of Infectious Disease Epidemiology, School of Public Health, Imperial College London, London, UK

**Keywords:** Infection attack rate, Cumulative incidence, Seroprevalence, Influenza, Serological survey, Cross-sectional study design, Longitudinal study design, Mathmatical modelling

## Abstract

**Background:**

Influenza infection does not always cause clinical illnesses, so serological surveillance has been used to determine the true burden of influenza outbreaks. This study investigates the accuracy of measuring cumulative incidence of influenza infection using different serological survey designs.

**Methods:**

We used a simple transmission model to simulate a typical influenza epidemic and obtained the seroprevalence over time. We also constructed four illustrative scenarios for baseline levels of antibodies prior and levels of boosting following infection in the simulated studies. Although illustrative, three of the four scenarios were based on the most detailed empirical data available. We used standard analytical methods to calculate estimated seroprevalence and associated confidence intervals for each of the four scenarios for both cross-sectional and longitudinal study designs. We tested the sensitivity of our results to changes in the sampled size and in our ability to detect small changes in antibody levels.

**Results:**

There were substantial differences between the background antibody titres and levels of boosting within three of our illustrative scenarios which were based on empirical data. These differences propagated through to different and substantial patterns of bias for all scenarios other than those with very low background titre and high levels of boosting. The two survey designs result in similar seroprevalence estimates in general under these scenarios, but when background immunity was high, simulated cross-sectional studies had higher biases. Sensitivity analyses indicated that an ability to accurately detect low levels of antibody boosting within paired sera would substantially improve the performance of serological surveys, even under difficult conditions.

**Conclusions:**

Levels of boosting and background immunity significantly affect the accuracy of seroprevalence estimations, and depending on these levels of immunity responses, different survey designs should be used to estimate seroprevalences. These results suggest that under current measurement criteria, cumulative incidence measured by serological surveys might have been substantially underestimated by failing to include all infections, including mild and asymptomatic infections, in certain scenarios. Dilution protocols more highly resolved than serial 2-fold dilution should be considered for serological surveys.

**Electronic supplementary material:**

The online version of this article (doi:10.1186/1471-2334-14-505) contains supplementary material, which is available to authorized users.

## Background

Influenza infection does not always cause clinical illnesses and the rate of non-clinical infection most likely varies from strain to strain [1]. Therefore, with the majority of surveillance systems based on clinical episodes, uncertainty regarding the number of unobserved infections dominated other epidemiological uncertainties during the 2009 pandemic [2].

Serological studies provide one option with which to resolve these uncertainties and a number were conducted (or at least initiated) during the 2009 H1N1 pandemic [3–5]. The rationale for conducting serological studies is straightforward as complimentary surveillance activity to traditional symptom-based and laboratory-based surveillance. Serological studies provide the alternative approach of monitoring immunity levels in a population and do not need to test people during a short period of time when they are symptomatic. In cross sectional serological studies, a single blood sample is drawn from members of the population and tested for the presence of high levels of antibodies to the virus of interest. In longitudinal serological studies, two or more samples are taken from members of the population and are tested for significant rises in antibodies.

In contrast to serology-based community studies, the measurement of influenza incidence in the community using PCR-based assays is not feasible because of the short time during which infected individuals shed virus. The intensity of sampling and testing would be prohibitively expensive. Serological studies also have advantages over symptom-based surveillance. Not all influenza-like-illnesses (ILI) are caused by influenza infection, nor does every influenza infection result in an influenza-like-illness. Despite the fact that not every infection results in increased antibody titres, it might be expected that assay-measured increases in effective antibody concentration are considerably less biased than symptom-based definitions such as ILI.

Despite these advantages of some alternative survey designs, serological surveys do suffer from a number of limitations. In particular, intuitively, two features of the population and strain affect the likely accuracy of a serological survey: levels of pre-existing antibodies to the strain of interest (most likely caused by cross-reactivity to prior circulating strains) and an inability of the virus to generate high levels of antibody boosting. Here, we investigate the impact of these two ecological features on the ability of two different study designs to estimate accurately the cumulative incidence of infection. Cumulative incidence based on seroepidemiological study is a measurement of seroprevalence, which quantifies the proportion of individuals whose serological specimens indicate seropositive against an infective pathogen. Unlike case prevalence that quantifies disease occurrences during a study period, seroprevalence quantifies antibody prevalence based on serological test that reflects the cumulative experience, past and recent infection, with an infectious agent.

## Methods

We used a parsimonious disease-dynamic model to make a deterministic prediction of seroprevalence at a given number of days after the introduction of a novel respiratory pathogen. From this, we simulated estimates of seroprevalence from the appropriate statistical model for either a cross-sectional or a longitudinal study, based on four illustrative scenarios for the baseline level of antibodies in the population and the degree of boosting after infection.

### Disease-dynamic model

The transmission process was modeled as a deterministic density-dependent susceptible-infected-recovery (SIR) model: A SIR model involves only three health states, namely, susceptible, infected, and recovered, in which the number of infected individuals (either at a specific instant or the cumulative occurrence) was the primary outcome. A density-dependent model simulates that the number of contacts is dependent of the susceptible population size as attack rate stays constant; whereas, demographic changes to the population such as births and deaths were not considered to be important here for the short period of interest. The model can be easily parameterised in terms of the reproductive number *R*[6] and the average time between generations of infection *T*_*g*_[7].

### Illustrative scenarios for baseline antibodies and boosting

We defined four different scenarios for baseline titres and boosting. The first scenario, Scenario A, was entirely theoretical and was used to demonstrate that both longitudinal and cross-sectional designs give unbiased estimates of seroprevalence under best-case assumptions. In Scenario A, we assumed that no individuals had detectible antibody titre and that all individuals underwent antibody boosts to 1:40 following infection.

Scenarios B and C were based on data from the Hong Kong Longitudinal Cohort Study [8]. Scenario B used titre values against A/California/4/2009(H1N1) and Scenario C used titre values against A/Perth/16/2009(H3N2). No PCR data was available for either of these scenarios, so prior to any sensitivity analyses, we assumed that titre increases of 2-fold or greater were actual infections but that we could only use rises of 4-fold or greater to reliably infer infection. All study protocols of Hong Kong Longitudinal Cohort Study were approved by The Institutional Review Board of The University of Hong Kong/Hospital Authority Hong Kong West Cluster.

Scenario D was based on a group of PCR-confirmed infections from 2009 in England and Wales for which serological assay results were also available [3]. Unfortunately, it was not possible to match baseline and follow-up titres at the level of the individual for this cohort. In this study, 1403 serum samples were collected in 2008 and 1954 serum samples were collected in 2009. We extracted the pre- and post-infection titre levels from the published paper, of which only those who had shown titre level rise were included. Then, since individuals’ boosting levels were unavailable, we estimated their boosting level as the most minimum possible according to the different combinations of pre- and post-infection titre values.

#### Model of immune responses

Haemagglutination-inhibiting (HI) antibody titres were represented by titre thresholds in the form of (<1:10, 1:10, 1:20, 1:40,..., 1:1280) for datasets from Riley and colleagues [8]; whereas, those from Miller and colleagues [3] were represented in (<1:8, 1:8, 1:16, 1:32,..., 1:1024). For mathematical convenience, we transformed both the baseline and post-infection antibodies onto non-negative integers, *y*, such that *y*=*log*_2_(*z*/*A*), where the actual titre threshold was 1:z and we assumed that <1:8 was equal to 1:4 (making *A*=4) and <1:10 was equal to 1:5 (*A*=5). On this scale, a four-fold difference or greater in titres corresponded to an increase of 2 or more in *y*.

The deterministic model provides a prediction of the cumulative incidence over time. We assumed that our serological study was of size *n*. We then used a simple statistical simulation model to generate the results of serological surveys. Each simulated survey was assumed to have drawn baseline blood samples at time *t*=0 and followup samples at time *t*=*t*_*f*_. We drew from the assumed baseline distribution of log titres for all *n* individuals in the simulated study. Although we considered many different values for *t*_*f*_, we never assumed more than a single follow-up sample was taken from any individual. The difference in cumulative incidences between times *t*=0 and *t*_*f*_ gave us the proportion of the population who were infected. Therefore, we randomly assigned each individual as infected or not based on that proportion. The follow-up log titre for those not infected was assumed to be the same as their baseline log titre. For those infected, we drew a random log boosting value from the assumed log boosting distribution, added that to their baseline log titre and recorded the resulting value as their follow-up log titre.

Based on the definitions of seroprevalence of different survey designs, the seroprevalence and estimated errors can be quantified as a function of pre- and post-infection antibody levels. Specifically, in the analysis of serial cross-sectional study design, we defined seroprevalence as the proportion of individuals in the population who were seropositive after excluding the proportion of individuals in the population who were seropositive at baseline [4]. Conversely, in the analysis of paired sera samples in longitudinal studies, seroprevalence was defined as proportion of individuals in the population that had a 2 unit of greater increase in log titre [8].

## Results

**Figure 1:**
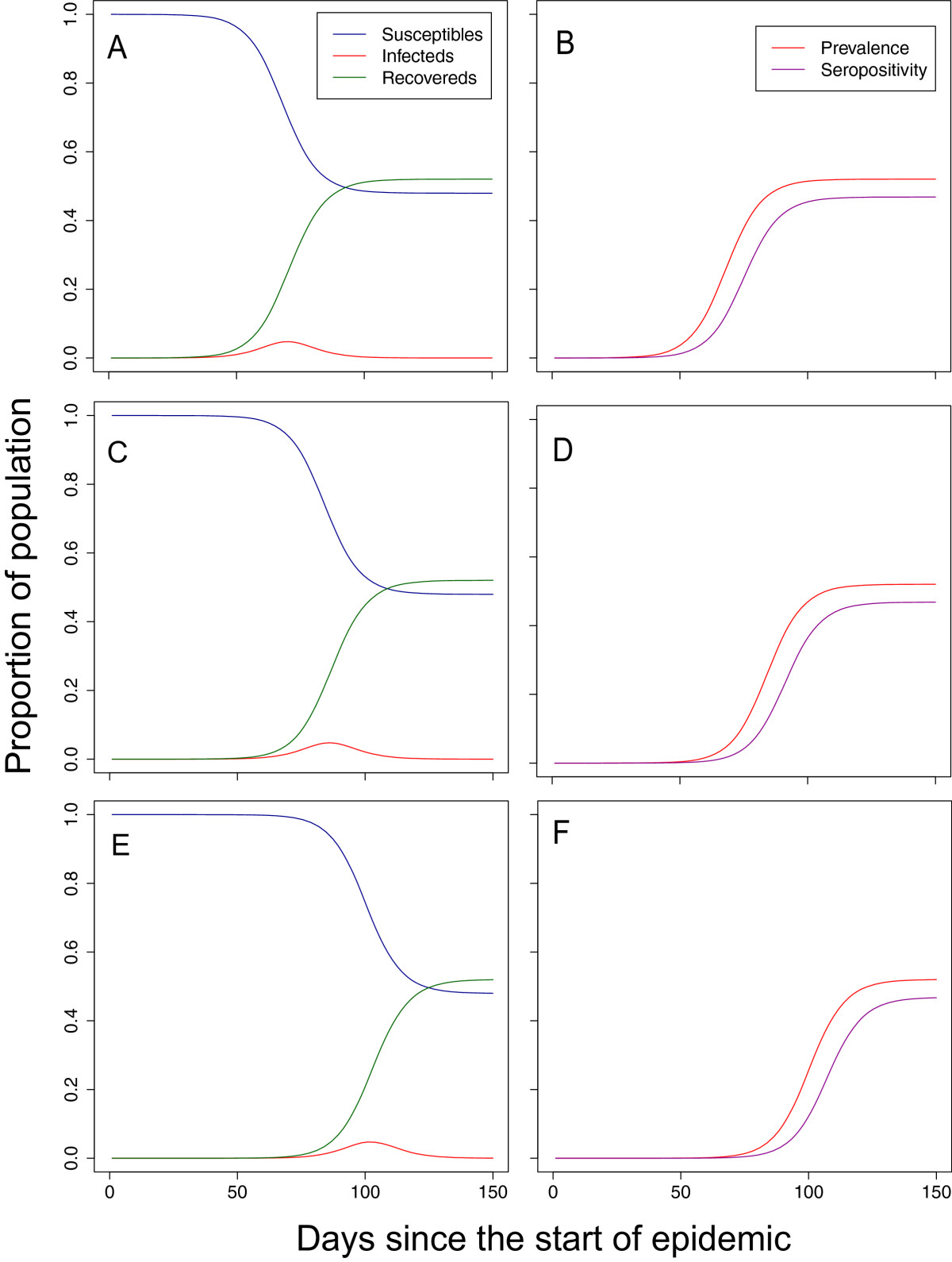
**Theoretical epidemic generated using an SIR model.** **LHS** numbers of susceptible, infectious and recovered individuals over time. **RHS** Incidence of infection and incidence of seroconversion (assuming 90% of individuals seroconvert after 7 days). All model solutions use a reproductive number of *R*=1.4, a mean generation time *T*_*g*_=2.6 days, and are seeded at time *t*=0 with a single infectious individual. Scenario A, parts **A** and **B**, occurred in a population size of 100,000 individuals. Scenario B, parts **C** and **D**, occurred in a population size of 1 million. Scenario C, parts **E** and **F**, occurred in a population size of 10 million.

**Figure 2:**
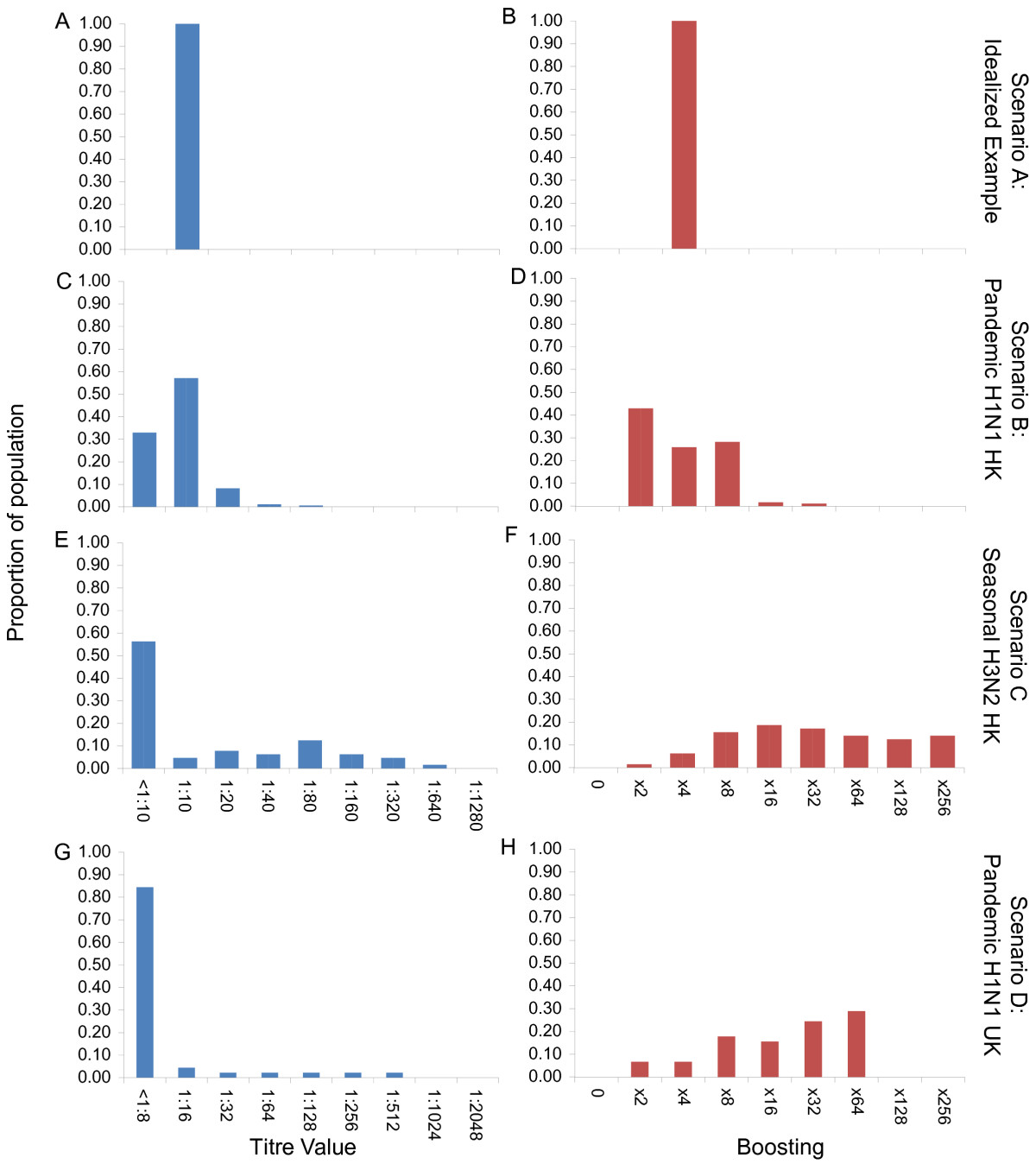
**Baseline titre level (LHS, blue) and boosting level (RHS, red) for the four different scenarios considered here: Scenario A, parts A and B; Scenario B, parts C and D; Scenario C, parts E and F; and Scenario D, parts G and H.** See Methods for details of data sources and assumptions for each scenario.

If estimates of the reproductive number *R* and the generation time *T*_*g*_ are available prior to the arrival of a new strain, disease-dynamic models can be used to anticipate that speed and hence aid the planning of serological studies. Specifically, it is useful to be able to predict the seroprevalence at some future point in time to ensure that studies are well-powered. For this simulation study, *R*=1.4 was chosen because it agrees with the estimated value in pandemic H1N1 influenza [9, 10] and seasonal influenza A [11, 12] in the community. Despite a relatively low transmissibility of *R*=1.4, peak numbers of infectious individuals in our illustrative epidemic (Figure 1A) occurred after just 60 days from the time of the importation that successfully initiated the local epidemic. The rapid timescale was driven by a short generation time of 2.6 days. The speed of epidemics predicted by the model scaled linearly with the generation time (not shown). Therefore, if the generation time were doubled, the delay from importation to peak numbers of infectious individuals would also double. The speed of the epidemic was sensitive to the size of population. When the population size was increased from 100,000 to 1 million (Figure 1C) and 10 million (Figure 1E), the speed of the epidemic slowed down considerably, but not linearly with population size. Not all infected individuals seroconvert after being infected. Even a relatively modest delay of 7 days [3] from infection to raised titres with a high seroconversion rate of 90% made a substantial impact of the time course of detectable infections (Figure 1B,D,F).There were significant differences among the baseline titre scenarios based on empirical data: B, C and D (Figure 2 LHS). Scenario B was based on empirical data for the 2009 pandemic strain of H1N1 infection inHong Kong. Under Scenario B we assumed that 67.1% (59.4%, 74.1%) of the population had detectable titre and within these individuals the geometric mean titre was 1:11 (1:10, 1:20). Scenario C was based on data from Hong Kong, but for seasonal H3N2. In these assays, 43.8% (31.4%, 56.7%) of individuals had detectable titre and of those the geometric mean titre was 1:62 (1:10, 1:320). Scenario D was based on pandemic H1N1 2009 in the UK and assumed that 15.6% (6.5%, 29.5%) of the population had detectable antibodies prior to the start of the epidemic and that the geometric mean titre of those that did have detectable antibodies was 1:35 (1:8, 1:218).There were also substantial differences in levels of boosting between the three scenarios based on empirical data (Figure 2 RHS). Average boosting under Scenario B was 4.8-fold (2.0-fold, 8.0-fold). Under Scenario C, average boosting was 27.8-fold (2.0-fold, 128.0-fold). Average boosting under Scenario D was even higher, based on data from PCR-confirmed cases of pandemic H1N1 infections in the UK, at 30.6-fold (2.4-fold, 64.0-fold). The apparent increased average boosting of Scenario C compared with Scenario B was driven by the right-tail of the distribution.

**Figure 3:**
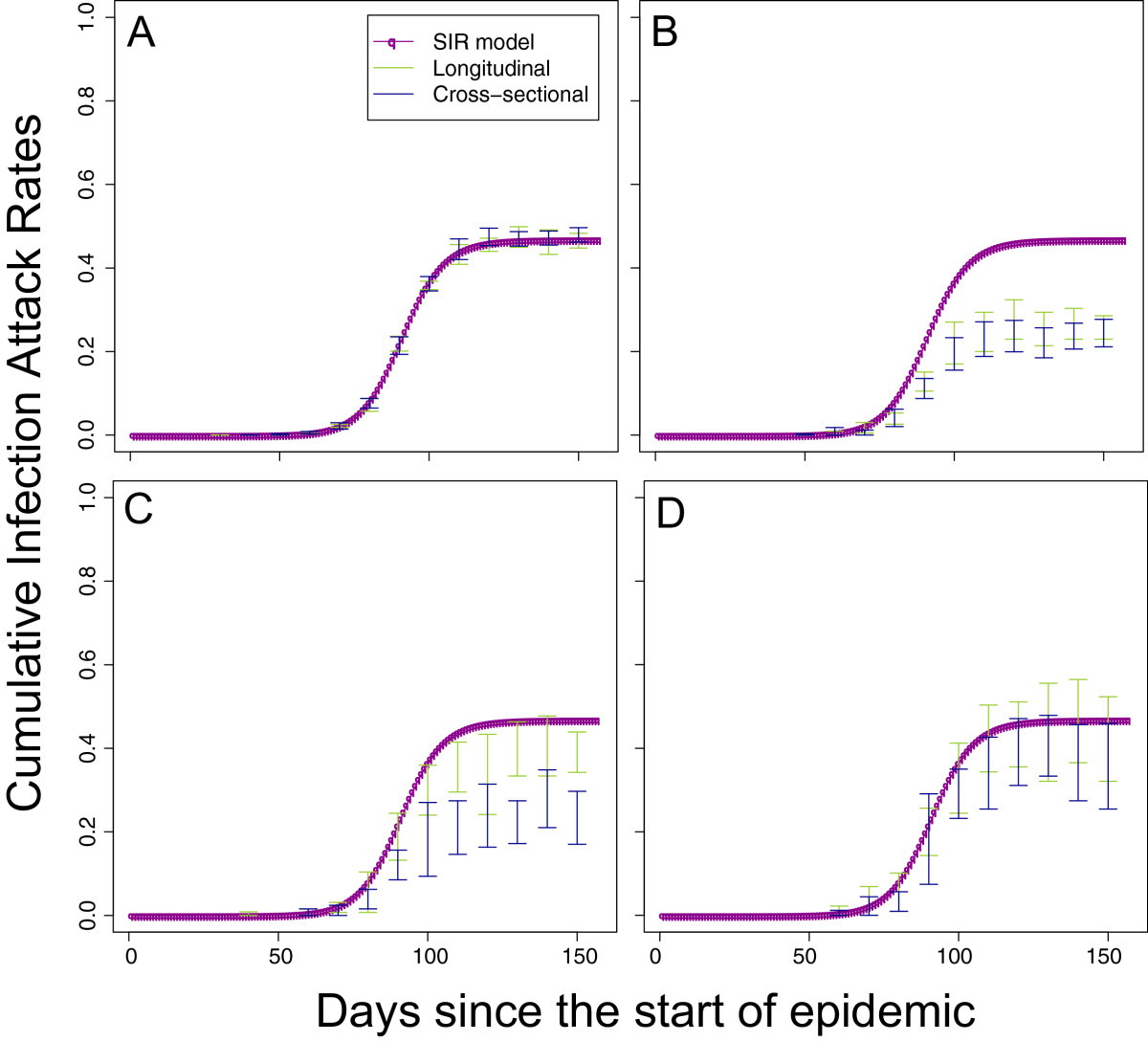
**Simulated study estimates of cumulative incidence of infection with population size 1 million.** Red line shows the SIR model cumulative incidence. Green line shows the 95% confidence intervals of cumulative incidence estimates from a longitudinal design and blue lines the 95% confidence intervals of cumulative incidence from a cross-sectional design. **A**,**B**,**C**, and **D** correspond to Scenarios A,B,C, and D (see Figure 2 and main text).

**Figure 4:**
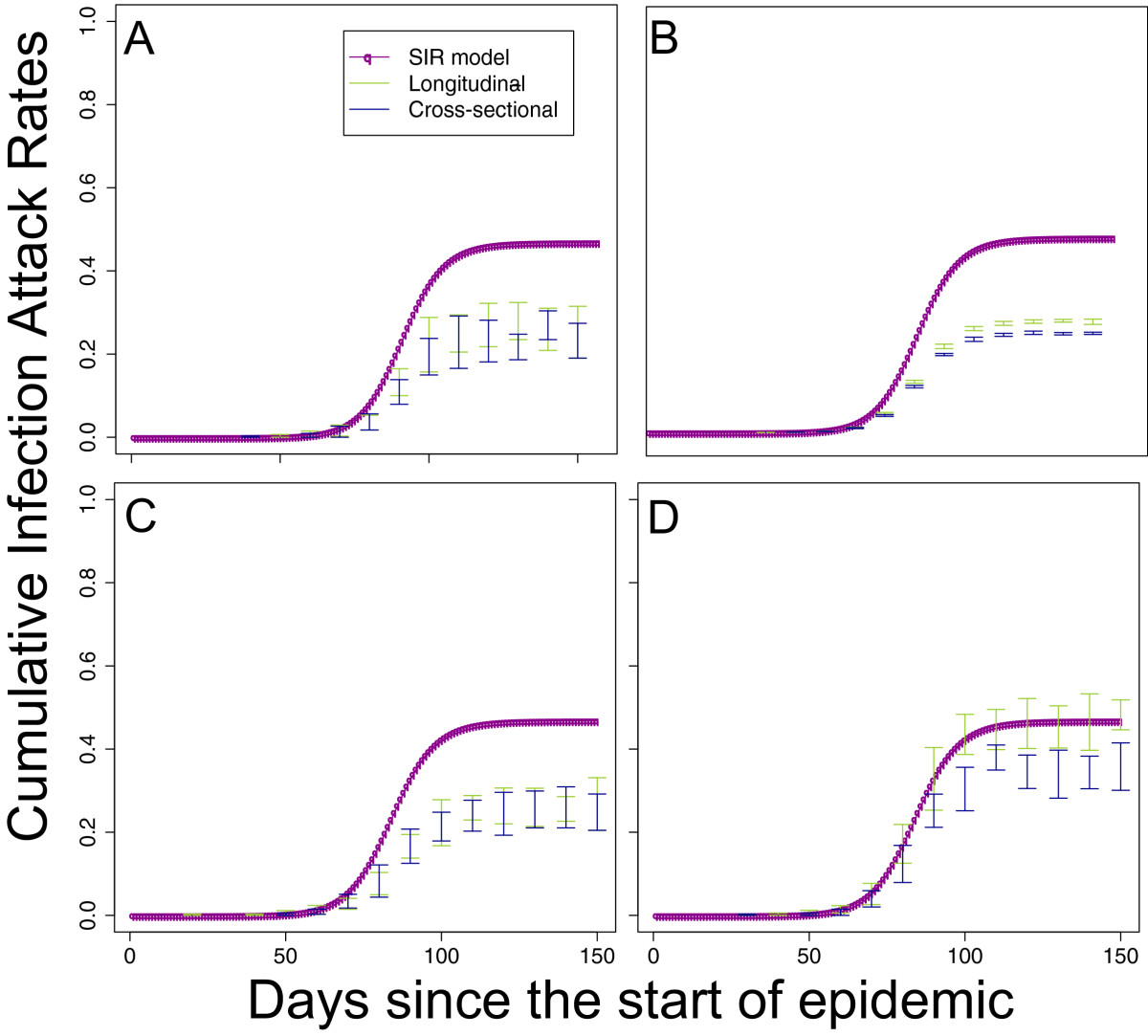
**Sensitivity analysis for worst performing scenario.** **A** shows estimates of cumulative incidence from longitudinal and cross-section study designs for Scenario B (pH1N1 from Hong Kong, identical to Figure 3B). The other 3 parts show sensitivity analyses for this scenario, with each alternate assumption applied separately to those used for part A. For **B**, the sampled size was increased 100 times. For **C**, we assumed that there was a zero-day delay between infection and our ability to detect infection. For **D**, we assumed that x2 increases in titre could be considered as indicators of infection.

Under the idealised Scenario A, as would be expected, there were no apparent biases in the simulated estimates of cumulative incidence under either the cross-sectional or longitudinal study design in a population of 1 million with a sample size of *n*=1,000 (Figure 3A). However, the same did not hold for the 3 scenarios based on empirical data (Figure 3B,C,D). Scenario B performed the worst, with both longitudinal and cross-sectional designs substantially underestimating the cumulative incidences because of the high levels of detectable antibody and low levels of boosting. Scenario C did somewhat better in that the degree of bias was limited for the longitudinal design but still substantial for the cross-sectional design. The improvement of Scenario C compared with Scenario B was because of the more consistent levels of boosting in Scenario C. Both longitudinal and cross-sectional designs performed well under the assumptions of Scenario D in which there were very high levels of boosting and very low levels of background immunity.In order to explore the reasons for the most substantial biases, we tested a number of alternate assumptions for the worst performing scenario, Scenario B (Figure 4). As might be expected, keeping the population size at 1 million but increasing the sampled size 100 times from 170 to 17,000 did little to reduce the amplitude of bias but did reduce the size of our confidence intervals substantially. Also, unrealistically eliminating the delay from infection to detectible antibodies did not alter the pattern of estimates from the study protocol, although the delay did shift the description of the epidemic forward in time. However, changing our assumption about the ability of serological assays to detect infections that results in low levels of antibody boosting did have a large effect on the simulated results from both longitudinal and cross-sectional study designs. Biases were substantially reduced for the cross-sectional study design and (apparently) eliminated completely for the longitudinal design (as shown by the reductions in the difference between the actual cumulative incidence and the estimated cumulative incidence in Figure 4D).

## Discussion

For influenza A, we used available data to define three illustrative scenarios for baseline titre values and boosting of titres following infection. We simulated cross-sectional and longitudinal serological studies based on these scenarios in order to assess the accuracy with which it was possible to measure the cumulative incidence of infection. We found that plausibly high levels of background immunity (perhaps due to cross-reactivity) and plausibly low levels of boosting following infection could introduce substantial biases to the estimates of cumulative incidence. Although biases were higher for cross-sectional study design than for the longitudinal study design in general, when levels of background immunity were low, there was little difference between the performances of the two designs. Sensitivity analyses indicated that an ability to detect infections from low levels of antibody boosting would substantially improve the performance of serological surveys, even under difficult conditions, i.e., when background titres are high and/or boosting after infection is low. Such conditions were observed in the elderly in Hong Kong during the 2009 pandemic.

The baseline-boosting scenarios used here were based on two different empirical study designs. Scenarios B and C drew on data from a longitudinal community-wide seroprevalence study [8]. Therefore, the boosting assumptions for these scenarios reflect accurately the distribution of changes in antibody state during the epidemic. However, no independent data exist with which to define infection in these data so it was not possible to tease out assay variation from low-levels of infection [13]. Conversely, the data used to define boosting for Scenario D are based on PCR-confirmed infections and therefore accurately describe boosting for the cohort of individuals from which these data were obtained [3]. However, because these samples arose from clinical cases, they likely reflect patterns of antibody boosting among a more severe subset of infections. One way to overcome these symmetric challenges in the different data sets would be to conduct a community-wide cohort study with intense virological sampling in addition to baseline and follow-up serology.

The main purpose of the deterministic model was to produce a realistic proportion of the population who are infected between two time points. Variations in transmission parameters, such as the reproductive number *R* and generation time *T*_*g*_ would be important for future survey design, but are not important for the interpretation of the simulated serological surveys.

We chose to simulate the dynamics within only a single homogeneously mixing population. Usually, serological studies of influenza in the community will be motivated by a whole set of questions of which estimating the cumulative incidence will only be one. A number of these other questions will likely relate to specific population subgroups. For example, there may be an over-representation of one age group than another in the clinical cases and it might be hoped that the serological study will help to resolve if the difference is being driven by differential rates of infection or by differential rates of becoming symptomatic. Also, having a higher proportion of school-aged children in the population would drive the epidemic to peak earlier than what shown in a homogeneous population. However, the conclusion regarding the effects of background and boosting titre levels toward the accuracy of seroprevalence measurement would have been the same if the model were age-stratified. The framework we describe here would still be useful in the design of field studies motivated by important subgroup questions as long as individual subgroups are treated as separate populations - so sample sizes and timings of follow-up would be chosen with specific types of individual in mind. Also, it would be straightforward to extend the transmission model to include multiple age groups and thus describe expected differences in the timing of peaks of infection between subgroups [4, 14].

The determination of cumulative incidence of infection within a population using serological studies are not without weaknesses. For instance, as noted, mild and asymptomatic infections may yield antibody titre below the level of minimum detection limit and seroconversion. In fact, a proportion of the pandemic H1N1 infections in 2009 were defined as seronegative following virologically confirmed infection [1, 3, 15]. Also antibody titres may be reduced in patients who were undergoing antiviral treatment [1]. Nevertheless, this model can be extended to explore the effects of these issues if antibody titre boosted between baseline and follow-up by these scenarios are known.

Interpretation of the results of haemagglutination inhibition (HI) test and microneutralization (MN) assays may further be complicated by vaccination. Often (although not always) self-reported vaccination status is available from longitudinal studies and not from cross-sectional studies. The simulation methods we have described here could be extended to incorporate this extra uncertainty where the vaccination status of individuals is not known but where the average rate across the population is known. Although this usually applies to cross-section studies, there is no reason the potential bias could not be assessed for both studies.

Our simulations (Figure 4) showed that being able to reliably detect small increases in antibody titre could substantially improve the accuracy of longitudinal serepidemiological studies when conditions are difficult: when background titres are high and boosting after infection is sometimes low. Although recently developed novel statistical methods are able to tease apart low levels of infection from measurement error [13], these rely on the use of a PCR-confirmed subset of data. As already mentioned, it may be difficult to obtain these data for a representative sample of the population. Therefore, we suggest that the potential reduction in bias from a more sensitive assay illustrated in these simulation results justifies trials of dilution protocols with higher resolution than 2-fold, especially for longitudinal studies.

## Conclusion

High levels of background titres and low levels of boosting affect estimates of cumulative incidence of influenza infection derived from seroepidemiological studies. When background immunity is high, simulated cross-sectional studies are particularly prone to higher biases. Otherwise, the two survey designs produce similar seroprevalence estimates in general. Assays capable of reliably detecting low levels of boosting after infection would greatly improve the performance of longitudinal studies when conditions are difficult.

## Authors’ original submitted files for images

Below are the links to the authors’ original submitted files for images. Authors’ original file for figure 1 Authors’ original file for figure 2 Authors’ original file for figure 3 Authors’ original file for figure 4
