# Supplementary figures and images for: Simulation-guided design of serological surveys of the cumulative incidence of influenza infection
Source: BMC Infect Dis. 2014 Sep 17;14:505. doi: 10.1186/1471-2334-14-505 (PMC4261848; doi:10.1186/1471-2334-14-505)

Proportion of population

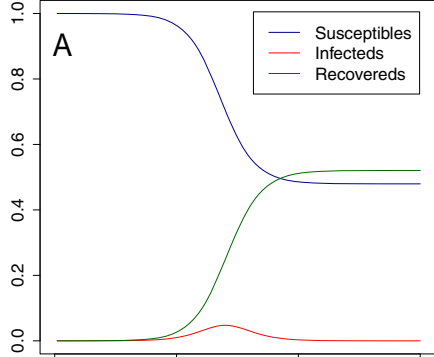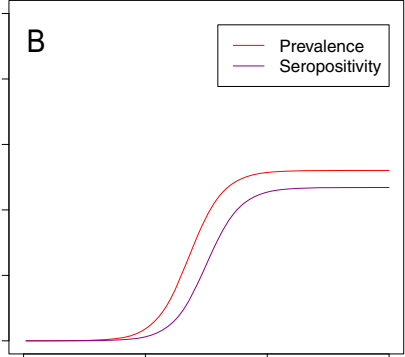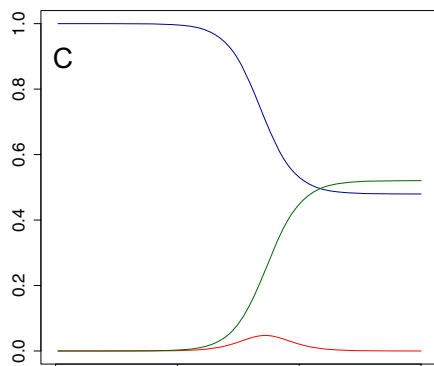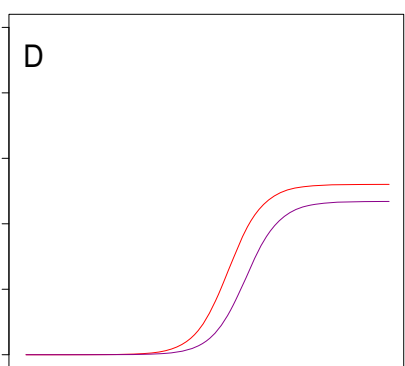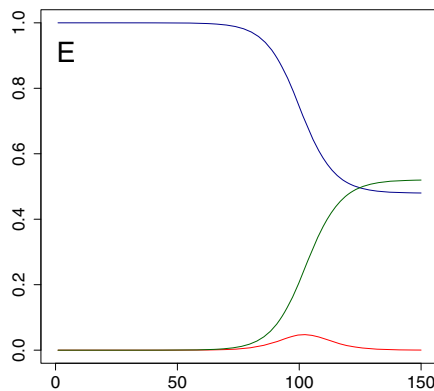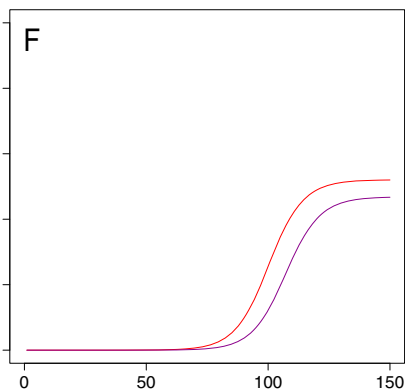

Days since the start of epidemic

Supplement: Supplementary file 1 — Authors’ original file for figure 1 [file 12879_2014_3836_MOESM1_ESM.pdf]

Cumulative Infection Attack Rates

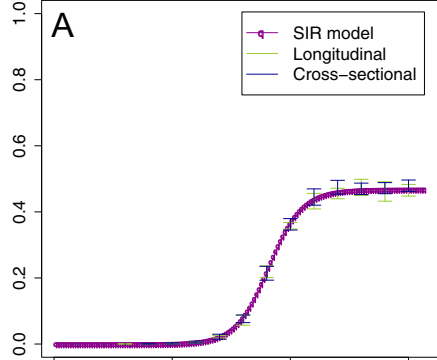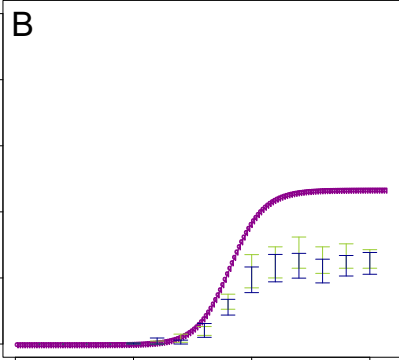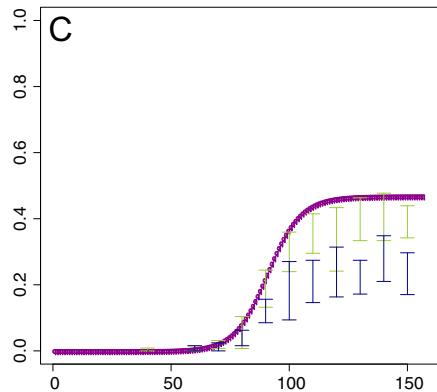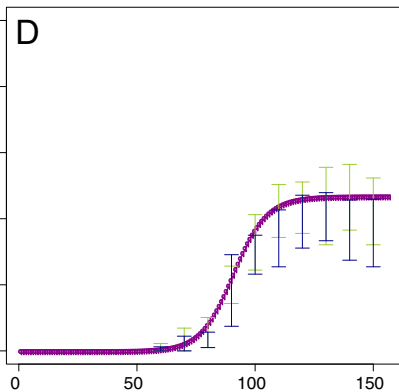

Days since the start of epidemic

Supplement: Supplementary file 3 — Authors’ original file for figure 3 [file 12879_2014_3836_MOESM3_ESM.pdf]

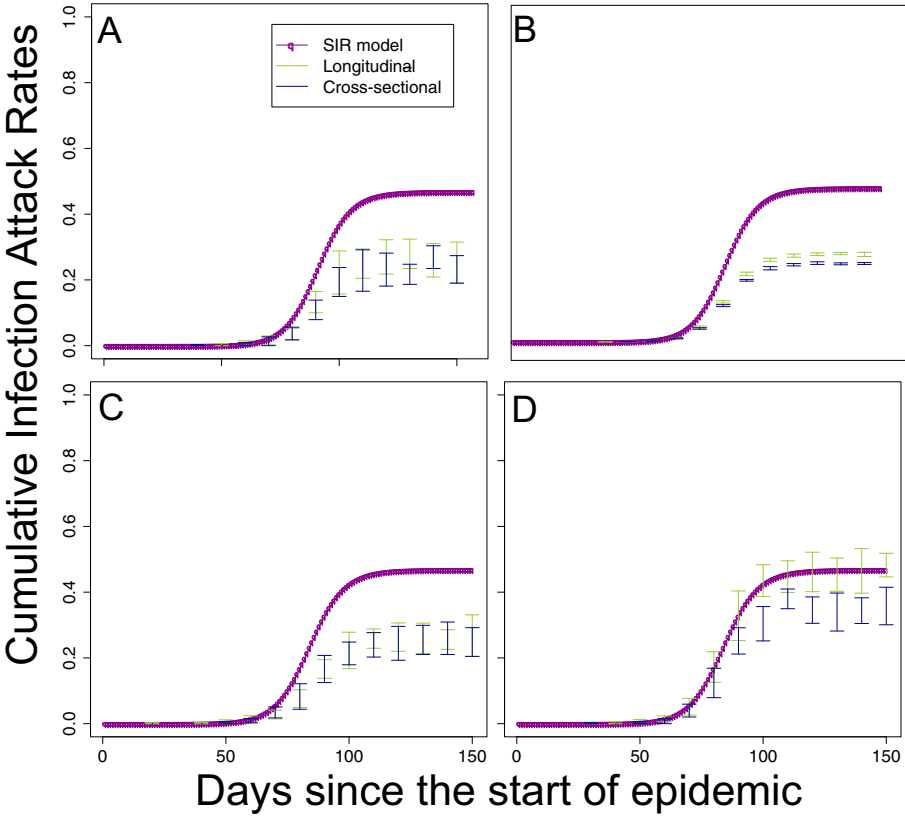

Supplement: Supplementary file 4 — Authors’ original file for figure 4 [file 12879_2014_3836_MOESM4_ESM.pdf]
