# Supplementary material for: Simulation-guided design of serological surveys of the cumulative incidence of influenza infection
Source: BMC Infect Dis. 2014 Sep 17;14:505. doi: 10.1186/1471-2334-14-505 (PMC4261848; doi:10.1186/1471-2334-14-505)
Supplement: Supplementary file 2 — Authors’ original file for figure 2 [file 12879_2014_3836_MOESM2_ESM.pdf]

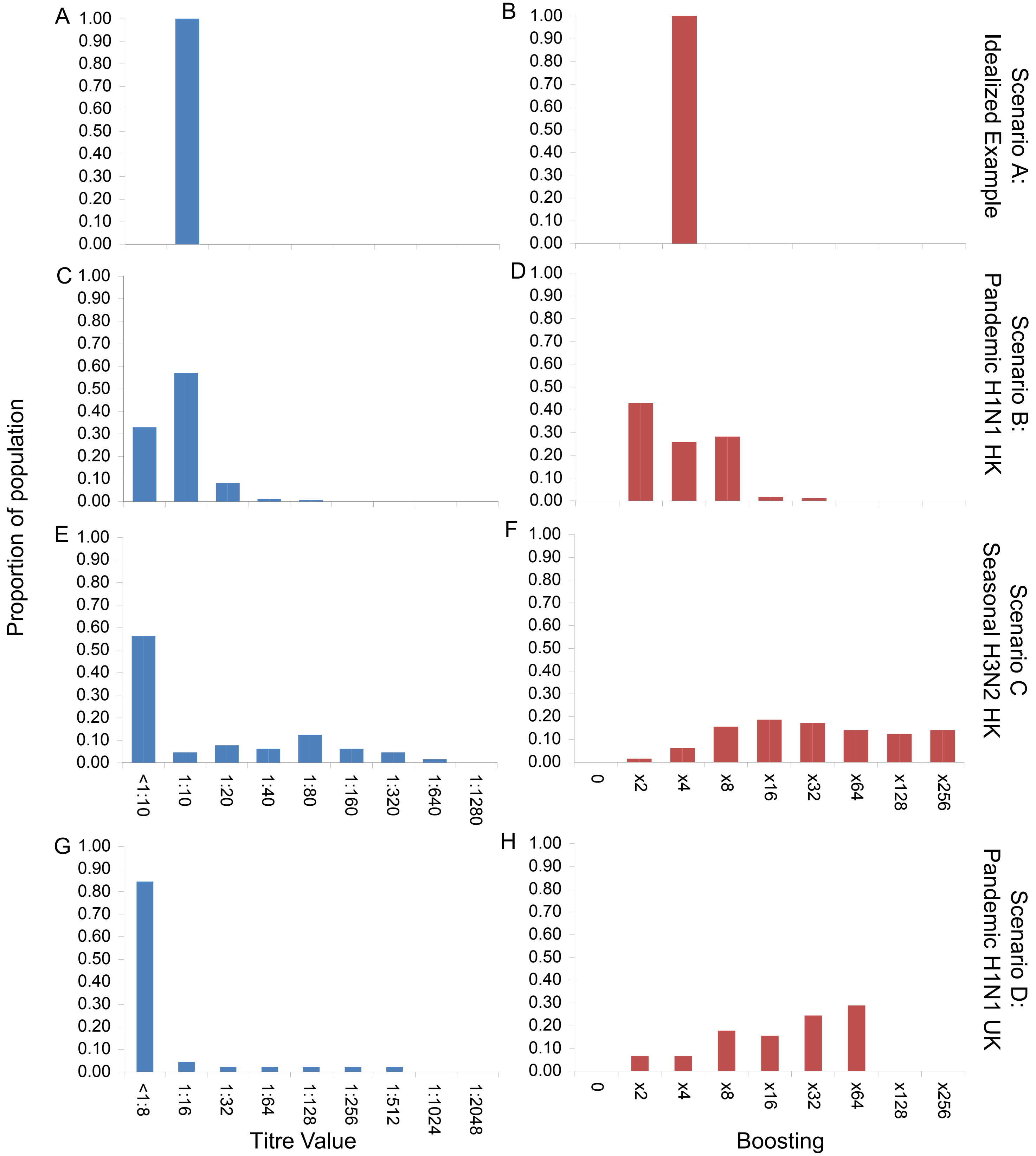

A
B
C
D
E
F
G
H
Scenario A: Idealized Example
Scenario B: Pandemic H1N1 HK
Scenario C Seasonal H3N2 HK
Scenario D: Pandemic H1N1 UK
Proportion of population
1.00
0.90
0.80
0.70
0.60
0.50
0.40
0.30
0.20
0.10
0.00
<1:10
1:10
1:20
1:40
1:80
1:160
1:320
1:640
1:1280
0
x2
x4
x8
x16
x32
x64
x128
x256
<1:8
1:16
1:32
1:64
1:128
1:256
1:512
1:1024
1:2048
Titre Value
Boosting
